# On the heterogeneity of the femoral enthesis of the human ACL: microscopic anatomy and clinical implications

**DOI:** 10.1186/s40634-016-0050-8

**Published:** 2016-07-13

**Authors:** Mélanie L. Beaulieu, Grace E. Carey, Stephen H. Schlecht, Edward M. Wojtys, James A. Ashton-Miller

**Affiliations:** School of Kinesiology, University of Michigan, 1402 Washington Heights, Ann Arbor, MI 48109 USA; Orthopaedic Research Laboratories, Department of Orthopaedic Surgery, University of Michigan, 109 Zina Pitcher Place, Ann Arbor, MI 48109 USA; MedSport, University of Michigan, Domino’s Farms, 24 Frank Lloyd Wright Drive, Lobby A, P.O. Box 391, Ann Arbor, MI 48106 USA; Biomechanics Research Laboratory, Department of Mechanical Engineering, University of Michigan, 2350 Hayward Street, GG Brown Building, Ann Arbor, MI 48109 USA

**Keywords:** Anterior cruciate ligament, Enthesis, Femur, Histology, Anatomy, Fibrocartilage, Tidemark, Shape

## Abstract

**Background:**

Most ruptures of the native anterior cruciate ligament (ACL) and ACL graft occur at, or near, the femoral enthesis, with the posterolateral fibers of the native ligament being especially vulnerable during pivot landings. Characterizing the anatomy of the ACL femoral enthesis may help us explain injury patterns which, in turn, could help guide injury prevention efforts. It may also lead to improved anatomic reconstruction techniques given that the goal of such techniques is to replicate the knee’s normal anatomy. Hence, the aim of this study was to investigate the microscopic anatomy of the ACL femoral enthesis and determine whether regional differences exist.

**Methods:**

Fifteen human ACL femoral entheses were histochemically processed and sectioned along the longitudinal axis of the ACL at 20, 40, 60, and 80 % of the width of the enthesis. Four thick sections (100 μm) per enthesis were prepared, stained, and digitized. From these sections, regional variations in the quantity of calcified and uncalcified fibrocartilage, the angle at which the ligament originates from the bone, and the shape profile of the tidemark were quantified.

**Results:**

At least 33 % more calcified fibrocartilage and 143 % more uncalcified fibrocartilage were found in the antero-inferior region, which corresponds to the inferior margin of the origin of the anteromedial ACL fibers, than all other regions (*P*s < 0.05). In addition, the anteromedial fibers of the ACL originated from the femur at an angle six times greater than did its posterolateral fibers (*P* = 0.032). Finally, average entheseal tidemark profiles correlated bilaterally (Pearson’s *r* = 0.79; *P* = 0.036), the most common profile being convex with a single re-entrant.

**Conclusions:**

Systematic regional differences were found in fibrocartilage quantity and collagen fiber attachment angles. The marked differences may reflect differences in the loading history of the various regions of the ACL femoral enthesis. These differences, which could affect the potential for injury, should also be considered when developing new ACL reconstruction approaches.

## Background

Anterior cruciate ligament (ACL) injuries occur at a rate of more than 250,000 incidences per year in the United States (Griffin et al. 2006) and present with long-term debilitative sequelae (Lohmander et al. 2007). For example, a review of the scientific literature revealed that 10–20 years after injury, 50 % of ACL-injured individuals developed knee osteoarthritis, including pain and functional impairment, regardless of whether the ACL was surgically reconstructed (Lohmander et al. 2007). Thus, better injury prevention programs and new approaches to ACL reconstruction need to be developed to reduce the considerable financial costs and negative health effects associated with these injuries (Riordan et al. 2013).

Most ruptures of the native ACL and ACL graft occur at, or near, the femoral origin (or “enthesis”) (Magnussen et al. 2012; Zantop et al. 2007a), with the posterolateral (PL) fibers of the native ligament being especially vulnerable during pivot landings (Beaulieu et al. 2015b; Lipps et al. 2013; Meyer et al. 2008). Hence, the microscopic anatomy of the ACL femoral enthesis, including regional differences, is of great clinical interest. Specifically, the amount of calcified (CF) and uncalcified fibrocartilage (UF) within an enthesis may reflect differences in the history of the forces experienced by the structure, and are known to vary within an enthesis (Evans et al. 1990; Evans et al. 1991). The ligament entheseal attachment angle is also clinically important because it can induce a strain concentration in the collagen fibers on the shorter (most proximal to interior angle) side of the tendon/ligament (Kim et al. 2011; Oh et al. 2013), with more acute angles inducing greater strains (Kim et al. 2011). Influencing the regional ligament entheseal attachment angle is the shape of the entheseal tidemark’s surface which, to our knowledge, has not hitherto been studied. A better understanding of these ACL entheseal parameters is important for two reasons. First, the propensity for the femoral enthesis of the PL fibers to be susceptible to injury during pivot landings (Beaulieu et al. 2015b; Lipps et al. 2013; Meyer et al. 2008) is unexplained. Hence, characterizing its anatomy may help explain injury patterns which, in turn, could help guide injury prevention efforts. Second, a better understanding of the native ACL anatomy could lead to improved anatomic reconstruction techniques, given that the goal of such techniques is to replicate the knee’s normal anatomy, restore its normal mechanics, and minimize incidences of graft failure (Fu et al. 2015). Additionally, this knowledge could be used for comparison purposes when assessing graft failure and its ligament-bone interface.

While several studies have described the native ACL femoral enthesis (Beaulieu et al. 2015a; de Abreu-e-Silva et al. 2015; Fujimaki et al. 2016; Iwahashi et al. 2010; Mochizuki et al. 2014; Sasaki et al. 2012), most have focused on macroscopic parameters such as footprint location, size, and/or shape (de Abreu-e-Silva et al. 2015; Fujimaki et al. 2016; Mochizuki et al. 2014). Fewer studies have focused on its microscopic characteristics (Beaulieu et al. 2015a; Iwahashi et al. 2010; Mochizuki et al. 2014; Sasaki et al. 2012). Results of these latter studies have described the enthesis as a combination of direct and indirect insertions, with the indirect fibers located proximal and posterior to the direct fibers (Iwahashi et al. 2010; Mochizuki et al. 2014; Sasaki et al. 2012) and extending to the posterior articular cartilage (Beaulieu et al. 2015a; Iwahashi et al. 2010). Fibrocartilage quantities have also been reported and suggest that regional differences may exist (Beaulieu et al. 2015a; Sasaki et al. 2012). However, heterogeneity in fibrocartilage quantity within the ACL femoral enthesis remains inconclusive because statistical comparisons were not performed. Furthermore, the ligament entheseal attachment angle — the angle at which the ACL attaches to the bone — was found to be more acute at the femoral enthesis than at the tibial enthesis (Beaulieu et al. 2015a). It is unknown, however, if it varies regionally within the femoral enthesis. Lastly, the entheseal tidemark profile — that is, the shape of the tidemark’s surface — is largely unknown, including its heterogeneity within an enthesis and between paired entheses (i.e., from left and right knees of donor).

The purpose of this study, therefore, was to investigate the microscopic anatomy of the ACL femoral enthesis and to determine whether or not there are regional differences within this structure. The primary null hypothesis was that there would be no regional difference in the relative area of CF, the average depth of UF, or the ligament entheseal attachment angle. The secondary null hypothesis was that all entheses, including entheses from the same donor, have similar tidemark profiles.

## Methods

### Specimen procurement & preparation

**Fig. 1:**
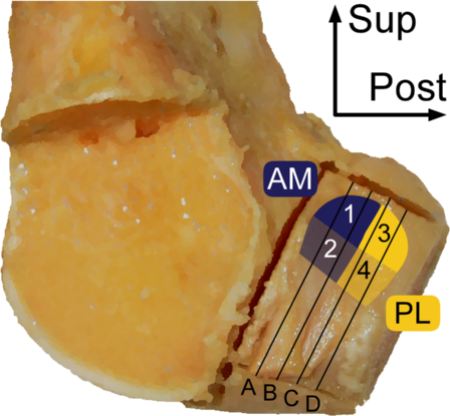
Excised femoral attachment site from a right distal femur showing the location of the tissue sections (*black lines*) processed and prepared, as well as the location of the regions of interest defined for histological analysis. A: 20 %; B: 40 %; C: 60 %; D: 80 % of the width of the enthesis. 1: antero-superior; 2: antero-inferior; 3: postero-superior; 4: postero-inferior regions. Regions 1–2 corresponded to the origin of the antero-medial (AM) fibers; meanwhile regions 3–4 corresponded to the origin of the postero-lateral (PL) fibers

This study was exempt from approval from the University of Michigan Institutional Review Board. To test the hypotheses, 15 unembalmed human knee specimens were harvested, including 14 paired and 1 unpaired specimens, from four male and four female donors (age = 52.1 ± 8.4 years; height = 1.70 ± 0.10 m; mass = 70.5 ± 15.9 kg; BMI = 24.1 ± 4.3 kg/m^2^) through the University of Michigan Anatomical Donations Program. The knee specimens were dissected down to the distal femur, proximal tibia, and ACL, and then fixed in 10 % neutral buffered formalin for 48 hours in 15 ° of flexion. A custom fixation device was built and used to hold the femur-ACL-tibia complex in this position, which allowed the ligament to maintain its natural twist and angle of attachment to each bone. With the ACL cut cross-sectionally at mid-substance, the femoral attachment site was extracted with an oscillating saw with a plunge blade (Bosch, Stuttgart, Germany) and trimmed with a diamond band pathology saw (EXAKT, Norderstedt, Germany). All tissue blocks were histochemically processed, which included defatting, dehydrating, clearing, and then embedding in methyl methacrylate (Beaulieu et al. 2015a). For each femoral enthesis, four thick sections (approximately 100 μm in thickness) were prepared, stained with toluidine blue, and digitized (resolution: 4,000 dpi). Sections were obtained parallel to the longitudinal axis of the ACL at 20, 40, 60, and 80 % of the width of the femoral entheses (Fig. 1).

### Quantitative analysis

For each digital image of the tissue sections, the relative area of CF, depth of UF, and ligament entheseal attachment angle were measured. CF relative area was quantified by outlining the CF tissue with a digital pen display (Cintiq 24HD w/ grip pen, Wacom, Kazo, Saitama, Japan) and dividing its area by the length of the enthesis. This enthesis length was defined as the length of the profile of the tidemark. Using the same tools, UF depth was measured at 500-μm intervals across the entire enthesis. Average depth, and not relative area, was selected as the method of choice for UF quantification for two reasons: first, the interface between this tissue and the adjacent distal tissue—dense fibrous connective tissue—is more ambiguous than between the CF and bone; and second, sampling UF depth at a constant interval is an established method (Beaulieu et al. 2015a; Evans et al. 1990). Lastly, the ligament entheseal attachment angle was quantified as the angle between a line parallel to the fibers of the ligament and a line of best fit (first order polynomial) to the profile of the digitized interface between the entheseal calcified and uncalcified tissue, also known as the “tidemark”. All measurements were made in ImageJ (Schneider et al. 2012). The average CF relative area and UF depth were calculated for four regions of interest of the femoral enthesis (Fig. 1): (1) antero-superior; (2) antero-inferior; (3) postero-superior; and (4) postero-inferior. Anatomically, regions 1–2 correspond to the origin of the anteromedial (AM) fibers, while regions 3–4 correspond to the origin of the PL fibers. These four regions were selected to allow for comparisons between the AM and PL fibers of the ACL, as well as between the superior and inferior margins. Lastly, the entheseal attachment angle was averaged over the two most anterior sections (Fig. 1, A–B), as well as over the two most posterior sections (Fig. 1, C–D), of each enthesis.

To measure the overall surface shape of the entheseal tidemark, the profile of the tidemark was quantified in each of the four tissue sections using custom Matlab code. Specifically, digital images of all sections were imported into Matlab, cropped to the width and height of the tidemark and resized so that all images were normalized to have the same width and height. Then, the tidemark profile was digitized and fit with a 5^th^ order polynomial. This order was selected because it was the lowest order polynomial that adequately represented the tidemark profile. For bilateral comparisons, the polynomial coefficients were averaged over all sections, resulting in an average polynomial, or “surface shape”, for each entheseal tidemark.

### Statistical analysis

A series of linear mixed-effects models were used to test whether regional differences existed in the amount of fibrocartilage and the magnitude of the ligament entheseal attachment angle within the femoral enthesis. The outcome variables for each model were CF relative area, UF depth, and entheseal attachment angle. The predictor variables for all models were the enthesis regions (coded as 1 = antero-superior, 2 = antero-inferior, 3 = postero-superior, and 4 = postero-inferior for CF and UF; and 1 = anterior sections and 2 = posterior sections for the entheseal attachment angle). Knee donor was included in the models to account for the correlation between specimens harvested from the same donor. To test whether the average polynomial describing the entheseal tidemark profile correlated bilaterally, the association between each polynomial coefficient was estimated with Pearson’s product moment correlation coefficient. One enthesis was excluded for this correlation analysis because its contralateral enthesis was not among the specimens included in this study. An alpha level below 0.05 indicated statistical significance.

## Results

**Fig. 2:**
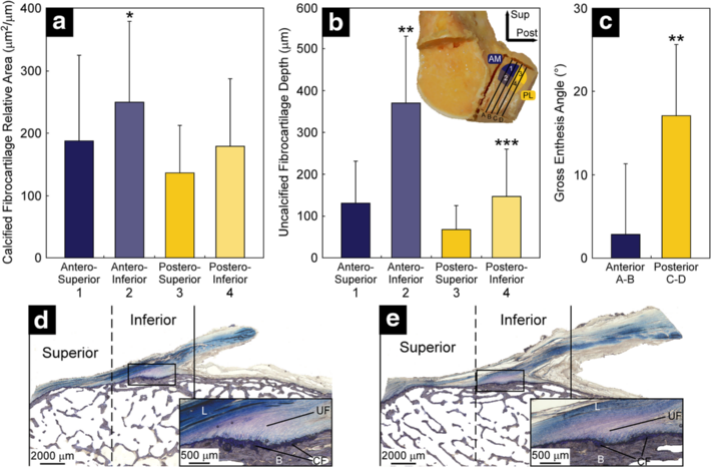
Mean and standard deviation of **a** relative area of calcified fibrocartilage and **b** depth of uncalcified fibrocartilage for each entheseal region of interest (1–4), as well as **c** ligament entheseal attachment angle of the anterior (A–B) and posterior (C–D) tissue sections presented for the femoral entheses. Example of **d** anterior (section B in Fig. 1) and **e** posterior (section C in Fig. 1) tissue sections of the ACL femoral enthesis of a representative specimen. *Insets*: High power views of tissue outlined in black showing four zones of tissue: ligamentous tissue (L), uncalcified fibrocartilage (UF), calcified fibrocartilage (CF), and bone (B). The symbol *denotes significantly greater than region 1 (*P* = 0.041), region 3 (*P* < 0.001), and region 4 (*P* = 0.020); **denotes significantly greater than all other regions (*P* < 0.001); ***denotes significantly greater than region 3 (*P* = 0.032)

The relative area of CF was significantly greater at the antero-inferior region of the ACL femoral enthesis than any other region (Fig. 2a, d, and e). This former region exhibited 33 % more CF than the antero-superior region (*P* = 0.041). Within the posterior sections, however, no significant difference in CF relative area was found (*P* > 0.050). Comparing the entheseal (anterior) sections that approximate the origin of the AM fibers of the ACL to those (posterior) sections that approximate the origin of the PL fibers, significant differences in CF relative area were found within the inferior margin. Specifically, the antero-inferior region exhibited 39 % more CF than did the postero-inferior region (*P* = 0.020).

The average depth of UF was also found to be heterogeneous, with significantly more UF at the antero-inferior region of the enthesis than in the other regions (Fig. 2b, d, and e). In fact, average UF depth in this region (which approximates the inferior margin of the origin of the ACL’s AM fibers), was 2.5 times greater than at the postero-inferior region (which approximates the inferior margin of the origin of the ACL’s PL fibers) (*P* < 0.001). Within the anterior sections, the inferior region exhibited 2.8 times more UF than the superior region (*P* < 0.001). Within the posterior sections, the inferior region exhibited 2.2 times more UF than the superior region (*P* = 0.032).

The ligament entheseal attachment angle was six-fold larger in the posterior sections than in the anterior sections (*P* < 0.001) (Fig. 2c), which correspond to the origin of the ACL’s PL and AM fibers, respectively.

**Fig. 3:**
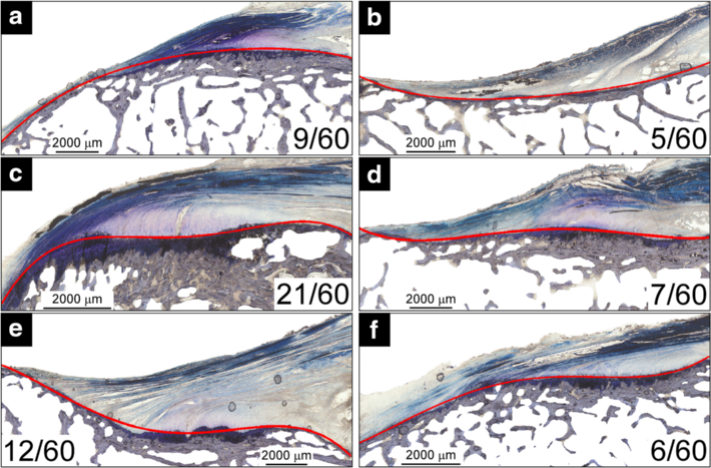
Examples of the six types of entheseal tidemark profiles: **a** convex; **b** concave; **c** convex with one re-entry; **d** concave with one re-entry; **e** half concave, half convex; **f** half convex, half concave. The proportion of sections classified as having a given tidemark profile is shown in the lower corner of each image. Type c was the most common tidemark profile

**Fig. 4:**
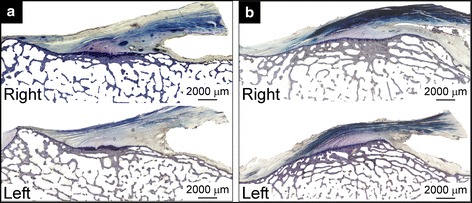
Examples of bilateral entheseal tidemark profile symmetry. Sections from the right and left ACL femoral entheses from **a** specimen #34578 and **b** specimen #34593

As for the profiles of the entheseal tidemarks, six profiles predominated (Fig. 3). The most common profile (21 out of 60 sections) was a convex profile with a single re-entrant (Fig. 3c). Most (8 out 9 sections) of the sections with a convex profile (Fig. 3a) were anterior sections of the entheses (Fig. 1, sections A–B). All the sections with a concave profile (Fig. 3b) were the most posterior section of the entheses (Fig. 1, section D). The occurrence of the other four types of entheseal profiles was more evenly distributed between the anterior and posterior entheseal sections (number of anterior/posterior sections per entheseal profile: 10/11 (Fig. 3c); 3/4 (Fig. 3d); 5/7 (Fig. 3e); 4/2 (Fig. 3f)). Lastly, the averaged entheseal tidemark profiles correlated bilaterally (fifth order coefficient, Pearson’s *r* = 0.786, *P* = 0.036) (Fig. 4 and Table 1). No other coefficients were significantly correlated bilaterally (*P*s > 0.050).

**Table 1:** Mean (standard deviation) coefficients of fifth-order polynomial fit to tidemark profiles of the left and right ACLs, including correlation coefficient and *P* values

Polynomial coefficients			Pearson correlation coefficient	*P* value
Order	Left	Right		
0	226 × 10^−17^ (550 × 10^−17^)	9.7 × 10^−17^ (440 × 10^−17^)	0.451	0.310
1	−238 × 10^−13^ (669 × 10^−13^)	−6.3 × 10^−13^ (499 × 10^−13^)	0.374	0.409
2	78 × 10^−9^ (287 × 10^−9^)	4.7 × 10^−9^ (207 × 10^−9^)	0.253	0.585
3	−4.5 × 10^−5^ (52 × 10^−5^)	−1.7 × 10^−5^ (45 × 10^−5^)	0.131	0.779
4	−22.0 × 10^−2^ (50 × 10^−2^)	−5.4 × 10^−2^ (65 × 10^−2^)	0.425	0.342
5	718.0 (290.6)	590.1 (303.2)	0.786	0.036

## Discussion

The present work investigated the microscopic anatomy of the ACL femoral enthesis and its regional differences, with the broader clinical aims of gaining evidence to improve the understanding of native ACL anatomy, help explain ACL injury patterns, and provide useful evidence that may help improve anatomic reconstruction techniques. Results indicate that the amount of fibrocartilage and the magnitude of the ligament entheseal attachment angle do indeed exhibit systematic regional heterogeneity in the femoral enthesis; hence the primary null hypothesis stated in the Introduction was rejected. The most fibrocartilage, whether CF or UF, was found at the antero-inferior region of the femoral enthesis, corresponding to the inferior margin of the origin of the ACL’s AM fibers. This heterogeneity in the amount of fibrocartilage at the femoral enthesis may reflect regional differences in the loading history applied to the enthesis, since the amounts of CF and UF are thought to positively relate to the tensile force applied to the bone and to the change in angle between the ligament and the bone to which it attaches, respectively (Benjamin et al. 1991; Evans et al. 1990; Evans et al. 1991). Hence, the inferior margin of the origin of the AM fibers may have been subjected to greater loading on a regular basis than other regions. This explanation is consistent with evidence that the ACL load is carried by only a few fiber groups at each knee flexion angle, with location of the fibers carrying the load varying with knee flexion angle (Mommersteeg et al. 1997). It is also consistent with results from a simplified finite element model of the pubovisceral muscle enthesis (Kim et al. 2011) which, like the ACL femoral enthesis (Beaulieu et al. 2015a), is a tensile structure that also arises from bone at an acute angle. The model revealed a strain concentration in the entheseal region where the shortest fibers of the tensile structure attach (Kim et al. 2011), corresponding to the inferior margin of the ACL femoral origin. So, why might the tensile loading be systematically greater at the inferior margin of the AM fibers than the PL fibers?

The answer may lie in their complementary roles in resisting anterior tibial translation and internal tibial rotation, respectively. Although all ACL fibers resist these loads to some degree, the AM fibers primarily resist anterior tibial translation (Christel et al. 2012), while the PL fibers primarily resist internal tibial rotation (Zantop et al. 2007b). In activities of daily living, such as walking and ascending/descending stairs, the knee mainly moves in the sagittal plane and primarily involves repetitive anterior tibial translation (Shelburne et al. 2004; Taylor et al. 2004). This results in the AM fibers systematically carrying more of the load than the PL fibers. Hence, the inferior margin of the AM fibers and their enthesis may be subjected to greater loads on a daily basis during activities of daily living than the PL fibers and their enthesis. This would explain the greater amount of CF that was found at the former location. However, the PL fibers’ femoral enthesis may be at greater risk of injury during pivot landings because they will be subjected to greater loads as the knee resists the internal tibial rotation that can occur during these landings (Zantop et al. 2007b). In fact, a recent finite element model of the ACL, to which impulsive loads consistent with a pivot landing were applied, revealed a strain concentration at the inferior margin of the PL fiber enthesis (Oh et al. 2013). Hence, there may be a shift in the location of the strain concentration at the femoral enthesis from the inferior margin of the AM fibers during activities of daily living to that of the PL fibers during athletic activities that involve large repetitive internal tibial torques, such as soccer, football, basketball, and volleyball, among others. It is possible that less fibrocartilage at the PL enthesis could make it vulnerable to injury during such pivoting activities.

Results of the present work corroborate the qualitative observation of Sasaki et al. (2012) that less fibrocartilaginous tissue exists in the superior regions (previously identified as “posterior” by Sasaki (2012)) of the femoral enthesis. These authors measured the combined depth of the CF and subchondral bone at the femoral enthesis (Sasaki et al. 2012), but made no regional statistical comparisons and did not independently assess the calcified and uncalcified fibrocartilaginous regions. They also categorized the enthesis as both a direct and indirect enthesis (Sasaki et al. 2012), termed fibrocartilaginous and fibrous, respectively, herein. The fibrocartilaginous portion of the femoral enthesis, which consists of four zones of tissue—ligamentous tissue, UF, CF, and bone (Schlecht 2012)—has been described as the central and inferior regions of the enthesis; while the fibrous portion, where the ligament originates directly from the bone without fibrocartilage (Schlecht 2012), has been said to comprise the most superior region of the enthesis (Sasaki et al. 2012). Since in this study some CF and, to a lesser extent, UF was found in this region, it is believed that the ACL femoral enthesis is more properly categorized as a fibrocartilaginous enthesis (Schlecht 2012).

A healthy fibrocartilaginous enthesis allows a gradual, instead of an abrupt, increase in stiffness in the structure from ligament to bone as it transmits mechanical loads from soft tissue to hard bone (Schlecht 2012). This is an important feature given that an abrupt increase in stiffness over such a junction is known to engineers to be more prone to failure due to the presence of a stress concentration. With regard to anatomic reconstruction techniques, evidence from animal models of graft-bone healing show that the four tissue zones characteristic of the fibrocartilaginous enthesis are not regenerated (Lu and Thomopoulos 2013). Instead, a layer of fibrovascular scar tissue is formed at the graft-tunnel interface. The healing process has been described as starting with disorderly fibrovascular tissue and evolving into an interface where the bone integrates with the superficial layers of the graft. Although this bone-graft integration enhances graft attachment strength, this insertion presents with inferior mechanical properties than the native ACL enthesis. In fact, Magnussen et al. (2012) reported that 74 % of ACL autografts failed near the femoral enthesis following a traumatic re-injury. These data, and more importantly the increased risk of developing knee osteoarthritis following ACL injury regardless of whether the ACL was reconstructed, suggest that new approaches to ACL reconstruction are desirable. The first step is gaining a better knowledge of the microscopic anatomy of the native ACL, especially its femoral enthesis. To that end, the results of the present study present an in-depth quantitative description of the ACL femoral enthesis and its heterogeneity in terms of cartilaginous tissue, attachment angle, and tidemark profile. These data may also be used as a basis for comparison for evaluating alternative methods to ACL reconstruction, such as stem cell therapy (Hao et al. 2016) and tissue engineering (Ma et al. 2012), among other applications.

The ligament entheseal attachment angle was significantly larger in the posterior rather than the anterior sections. This angle difference may be attributed to differences in entheseal profile between anterior and posterior sections since the most convex and concave profiles were found in the anterior and posterior sections, respectively. To our knowledge, the entheseal attachment angle and its regional differences have not been reported before. According to the simplified finite element model of the pubovisceral muscle enthesis, the entheseal attachment angle is inversely related to the strain concentration magnitude in the inferior margin (Kim et al. 2011). This suggests that, within the inferior margin of the femoral enthesis, the anterior region may experience a greater strain concentration than the posterior region, at least in the knee position examined herein (15 ° of knee flexion). This may partly explain the greater amount of fibrocartilage in the antero-inferior region than in the postero-inferior region. Conversely, the greater attachment angle in the posterior enthesis section is, in part, caused by the concave surface profile there; this may be an architectural mechanism to reduce the entheseal strain concentration in that region. This systematic variation in the angular orientation of the fibers forming the ACL femoral enthesis should be taken into consideration as new ACL reconstruction approaches are explored and developed. Although the location of the femoral tunnel has been investigated extensively (Xu et al. 2016), its direction has not, to our knowledge. This is an important factor to consider because the direction of the tunnel influences the angle of insertion of the collagen fibers in the graft. It remains to be determined, therefore, whether selecting a femoral tunnel direction that maximizes the attachment angle should be recommended given that acute angles present with strain concentrations of greater magnitudes (Kim et al. 2011).

**Fig. 5:**
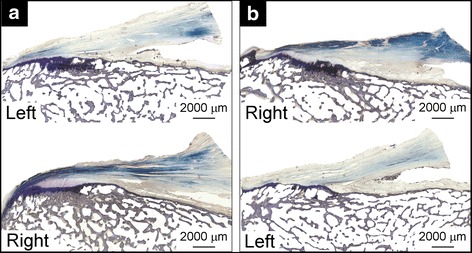
Histological images of **a** anterior sections (Fig. 1, B) and **b** posterior sections (Fig. 1, C) showing local concentrations in calcification (*top*) and the corresponding sections in the paired enthesis (*bottom*) from specimen #34602

The secondary null hypothesis that the tidemark of all entheses, including paired entheses, would have the same general surface shape was rejected. Several different tidemark architectural profiles were observed, varying from generally convex to concave as one moved from anterior to posterior sections. In addition, the average tidemark profiles, or “tidemark surface shapes”, were more strongly correlated bilaterally within than between individuals. These findings are not surprising given that entheseal surface shape is determined by the history of the tensile forces applied to the enthesis by the ligament during puberty (Gao and Messner 1996). Assuming that the donors of the knee specimens tested in this study subjected their ACL femoral entheses to different loading histories, one might expect the variations in entheseal surface shape to be greater between individuals than bilaterally within an individual, which is indeed what we found. Interestingly, some images exhibited local concentrations in calcification (Fig. 5) reminiscent of nascent Pellegrini Stieda known to form in other knee ligaments (Wang and Shapiro 1995). It remains to be seen whether these regions are evidence of local remodeling within injured fibrous regions.

The present study was not without limitations. First, ACL entheseal tissues were harvested from older donors. Although fibrocartilaginous entheses, such as the ACL femoral enthesis, can be affected by age-related degenerative changes (Villotte and Knüsel 2013), no evidence exists to suggest that these changes would affect one region of the enthesis differently than another. Second, the physical activity history of our specimens’ donors was unknown. We cannot assume, therefore, that our results can be generalized to the young, active population that suffers the most ACL injuries (Kobayashi et al. 2010). Third, our method to quantify the entheseal tidemark profiles was applied to two-dimensional images—histological sections—to represent a three-dimensional (3D) surface. Future work should utilize 3D imaging, such as micro computed tomography (CT), to obtain a 3D representation of the entheseal surface. Such a method would ‘connect the dots’ by revealing in more detail how the tidemark shape varies across the enthesis.

## Conclusions

In summary, most calcified and uncalcified fibrocartilage was found at the antero-inferior region of the femoral enthesis. The ligament entheseal attachment angle was more acute in the anterior than the posterior sections. Finally, although the characteristic shape of the femoral entheseal tidemark varied across individuals, some bilateral similarity was found within individuals. The marked differences may reflect differences in the loading history of the various regions of the ACL femoral enthesis. These differences, which could affect the potential for injury, should also be considered when developing new ACL reconstruction approaches.

## Abbreviations

3D, three-dimensional; ACL, anterior cruciate ligament; AM, anteromedial; CF, calcified fibrocartilage; CT, computed tomography; PL, posterolateral; UF, uncalcified fibrocartilage.
